# Enhanced enzymatic sugar production from corn stover by combination of water extraction and glycerol-assisted instant catapult steam explosion

**DOI:** 10.1186/s40643-024-00739-7

**Published:** 2024-03-14

**Authors:** Fengqin Wang, Hongli Dong, Weiwei Yu, Yinling Gao, Guotao Mao, Yanxia An, Hui Xie, Andong Song, Zhanying Zhang

**Affiliations:** 1 Key Laboratory of Enzyme Engineering of Agricultural Microbiology, Ministry of Agriculture, College of Life Science, Henan Agricultural University, Zhengzhou, 450046 People’s Republic of China; 2 College of Food Science and Technology, Henan Agricultural University, Zhengzhou, 450002 People’s Republic of China; 3 School of Mechanical, Medical and Process Engineering, Centre for Agriculture and the Bioeconomy, Queensland University of Technology, Brisbane, QLD 4000 Australia

**Keywords:** Glycerol, Steam explosion, Enzymatic digestibility, Water extraction, Lignin

## Abstract

Glycerol-assisted instant catapult steam explosion (ICSE) of lignocellulose is an effective pretreatment method for enhancing sugar production compared to glycerol-free ICSE. In this study, glycerol-assisted ICSE of corn stover was studied in order to understand the reaction mechanisms and further optimize the process. Results showed that water extraction of corn stover prior to ICSE reduced pseudo-lignin formation. The combination of water extraction and glycerol-assisted ICSE led to the formation of lignin with a lower molecular weight (Mw) of 2851 g/mol than 3521 g/mole of that from the combination of water extraction and glycerol-free ICSE. ^1^H-^13^C NMR analysis revealed that glycerol likely reacted with lignin carboxylic OHs through esterification while etherification of aliphatic OHs was not observed in ICSE. These lignin analyses indicated that glycerol protected lignin from condensation/repolymerization during glycerol-assisted ICSE. Enzymatic hydrolysis results showed that without water extraction increasing glycerol usage from 0.2 kg/kg stover to 0.4 kg/kg stover improved glucan digestibility to 78% but further increase to 0.5 kg/kg stover reduced glucan digestibility. In addition, at the glycerol usage of 0.2–0.4 kg/kg stover, washing of pretreated stover for removal of glycerol and other biomass-derived compounds did not improve glucan digestibility compared to unwashed ones. Combination of water extraction and glycerol-assisted ICSE led to a high glucan digestibility of 89.7% and a total glucose yield of 25.5 g glucose/100 g stover, which were 30.1% and 7.5 g/100 g stover higher than those derived from glycerol-free ICSE of stover, respectively. Since glycerol is a low-cost carbon source, the resulting enzymatic hydrolysate that contained both glucose and glycerol may be directly used to produce bioproducts by microbial fermentation.

**Graphical Abstract:**

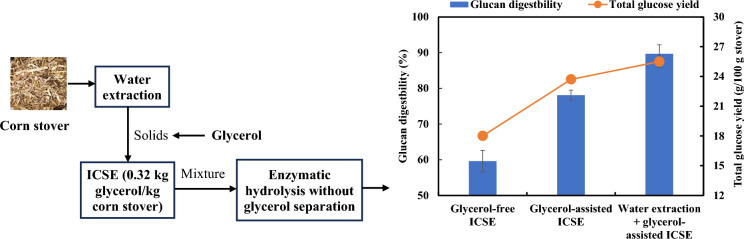

**Supplementary Information:**

The online version contains supplementary material available at 10.1186/s40643-024-00739-7.

## Highlights

- Glycerol-assisted ICSE of corn stover improved enzymatic glucose production.
- Water extraction prior to ICSE further enhanced sugar production.
- Enzymatic hydrolysis was not affected by glycerol from glycerol-assisted ICSE.
- Sequential water extraction and ICSE led to a high glucan digestibility of 89.7%.
- Glycerol likely had a multiple role in ICSE and enzymatic hydrolysis.

**Supplementary Information:**

The online version contains supplementary material available at 10.1186/s40643-024-00739-7.

## Introduction

Corn stover is an abundant renewable bioresource for producing biofuels and biochemicals. Like other lignocellulosic biomass, corn stover mainly consists of cellulose, hemicellulose and lignin. Due to the recalcitrant nature of lignocellulosic biomass, pretreatment is a prerequisite to improve fermentable sugars production by enzymatic digestion. Many methods, such as dilute acid, soda, ionic liquid, organosolv and steam explosion pretreatments have been reported for pretreatment of lignocellulosic biomass (Banu et al. 2021; Zhao et al. 2022). Of these methods, steam explosion is attractive because it is rapid and can be operated with or without the use of catalysts and/or solvents (Wang et al. 2020b; Yu et al. 2022).

Instant catapult steam explosion (ICSE) is a more powerful and effective steam explosion method than the conventional ones because of the relatively short explosion time of < 0.1 s (Yu et al. 2012). Previously, ICSE has been used to pretreat corn stover for enhancing glucan digestibility for sugar production (Liu et al. 2014), to disrupt microalgal cells for improving lipid extraction (Cheng et al. 2015), to treat feed corn stalk for degradation of toxin (aflatoxin B1) (Xie et al. 2020), to isolate high quality cellulose from rice straw (Gou et al. 2018) and to prepare high-valued bovine bone powder (Qin et al. 2022). In order to improve the pretreatment effectiveness for sugar production, ICSE was also combined with solvents, such as ionic liquids and glycerol to pretreat lignocellulosic biomass (Liu et al. 2016a; Wang et al. 2020a).

In the authors’ previous study, a glycerol-assisted ICSE process was developed to pretreat corn stover to improve sugar production (Wang et al. 2020a). The use of glycerol improved surface roughness of pretreated corn stover and might also protect lignin from condensation compared to the ICSE without glycerol (Wang et al. 2020a). As a result, the glycerol-assisted ICSE pretreatment at a glycerol/corn stover mass ratio of 1:1 improved glucan digestibility and glucose yield by ~ 28% and ~ 45%, respectively (Wang et al. 2020a). Although glycerol is a low-cost solvent and relatively friendly to cellulases used in enzymatic digestion compared to other solvents such as ionic liquids (Zhang et al. 2015), the use of a high loading of glycerol would increase the operational costs associated with biomass wash and solvent recovery, making the process economically less attractive.

Glycerol is a biodegradable carbon source and can be utilized alone or in combination with sugars for biosynthesis of bioproducts, such as microbial oils, mannitol and citric acid, by many microorganisms (Hassanpour et al. 2019; Kumar et al. 2021; Rakkitkanphun et al. 2021; Yoshikawa et al. 2014). Further reducing glycerol loading in ICSE may lead to the development of a process with which separation and recovery of glycerol are unnecessary and pretreated biomass can be enzymatically hydrolyzed in the presence of glycerol. However, the effect of further reducing glycerol loading on the pretreatment effectiveness of ICSE is still unknown.

In addition to the lignocellulosic biomass components, corn stover also has a relatively high content of water and/or ethanol extractives, which are nonstructural components, such as soluble sugars, phenolics and waxes (Li et al. 2016). The reported content of the extractives ranged from 7 to 23% (Huber et al. 2006; Li et al. 2016; Liu et al. 2013). The high content of extractives could have negative effects on pretreatment effectiveness (e.g., hemicellulose removal) and enzymatic digestibility of resulting biomass for glucose production as previously reported with dilute acid and liquid hot water pretreatments of corn stover, rice straw and sugarcane bagasse (Li et al. 2016; Zhang et al. 2016b). In terms of steam explosion pretreatment, it was reported that removal of water-soluble extractives from softwood barks and olive tree pruning also improved subsequent sugar production (Ballesteros et al. 2011; Franko et al. 2018). Many studies (if not all) on the effects of extractives on pretreatment and enzymatic digestibility have been undertaken based on the use of aqueous systems or steam. In the presence of glycerol in pretreatment systems, the effect of extractives has not yet been investigated.

The present study aimed to reduce glycerol loading in ICSE pretreatment, further understand the role of glycerol in pretreatment and enzymatic digestion, as well as develop an integrated process for direct enzymatic digestion without wash to remove glycerol. In this study, corn stover was pretreated at a solid/liquid mass ratio of 1:0.5 with the glycerol/water mass ratios varied from 0:5 to 5:0. The ICSE of corn stover was also compared with ICSE of water-extracted corn stover to understand the effect of water extraction on pretreatment and enzymatic digestibility. Lignin was extracted after enzymatic digestion and characterized to understand the pretreatment mechanisms. Finally, enzymatic hydrolyses of both washed and unwashed solid residues were compared in terms of glucan digestibility and sugar yield. The present study not only led to the development of a glycerol-assisted ICSE with significantly reduced usage of glycerol but also very useful information in understanding the pretreatment mechanism and selection of biomass processing approaches (water extraction or not, wash or not, co-utilization of glycerol and sugars or not, etc*.*).

## Materials and methods

### Raw materials

Corn stover was collected from a local corn field in Zhengzhou (Henan province, China). The corn stover was air dried and milled by a cutter grinder and passed through a sieve with an aperture size of 0.3 cm and was then stored at room temperature in a sealed container. Glycerol (≥ 99.7 wt%) and a commercial cellulase (CTec3 with protein concentration of 99.2 mg/mL and cellulase activity of 120 FPU/mL) were purchased from Tianjin Fuyu Fine Chemical Co. Ltd. and Novozymes (China) Biological Technology Co. Ltd., respectively.

### Extraction of corn stover by water

The milled corn stover sample was dried at 45 ℃ to a constant weight prior to use. 150 g of dried corn stover sample were mixed with deionized water at a ratio of solid to liquid 1:20. The weight of the mixture was recorded, followed by heating the mixture to 95 ℃ and incubation for 2 h with intermittent stirring every 10 min. At the end of incubation, the mixture was cooled to room temperature and weighed. Fresh water was added to the mixture to compensate the water loss during incubation. The mixture was then filtered, and the filtrate was collected for sugars analysis. The solid residue was then washed with water till the wash solution became colorless. The washed solid residue was dried at 45 °C to a constant weight.

### Glycerol-assisted ICSE pretreatment

Corn stover and water-extracted corn stover samples (100 g) were pretreated by ICSE at solid/glycerol/water mass ratios of 1:0.0:0.5, 1:0.2:0.3, 1:0.3:0.2, 1:0.4:0.1 and 1:0.5:0.0, respectively. The trials were recorded as 0:5, 2:3, 3:2, 4:1, 5:0 based on the ratio of glycerol and water, respectively. Due to the high viscosity of glycerol, for the trial of 5:0, glycerol was preheated to 90 °C in a water bath before it was mixed with corn stover sample. For other trials, liquid solutions were firstly prepared by mixing glycerol and water at required ratios, followed by mixing corn stover samples with liquid solutions at required ratios. All the mixtures were stored in self-sealed plastic bags overnight, followed by loading the mixtures to the preheated chamber (400 mL) of an ICSE reactor (QBS-80 SE, Hebi Gentle Bioenergy Co. Ltd., China) (Yu et al. 2012). The pressure of the chamber was increased to 2.0 MPa (corresponding 212 ℃) within 2–3 s by injection of steam with a pressure of 2.7 Mpa and maintained at 2.0 MPa for 2.5 min. Then the piston driving device was triggered to release pressure, resulting in an intense explosion within 0.1 s (Wang et al. 2020a). After ICSE, each of the pretreated sample (slurry) was collected, well-mixed and stored at 4 ℃ until further processing and analysis. ICSE was conducted in triplicate.

Processing the pretreated corn stover samples for determination of the water content in liquid fraction, water-soluble sugars and byproducts, composition of the solid fractions and glucan digestibility of the solid fraction followed the methods described in the authors’ previous publication (Wang et al. 2020a).

### Enzymatic digestion of pretreated corn stover samples

Enzymatic digestion was performed in 20 mL scintillation vials containing pretreated corn stover sample (washed or unwashed) at a glucan loading of 3 wt%, 0.05 M citrate buffer (pH 4.8), 0.02 wt.% sodium azide (to prevent the growth of microorganism), and cellulase at a dosage of 20 FPU/g glucan. The digestion was conducted at 50 °C in a rotary incubator with a shaking speed of 150 rpm. Samples were withdrawn at different time intervals for sugars analysis by high-performance liquid chromatography (HPLC). Enzymatic digestion was conducted in triplicate.

### Recovery of lignin for characterization

After enzymatic digestion, the solid residues were collected by centrifugation, washed with deionized water till colorless, and freeze-dried. 20 g of solid residues were milled with a planetary ball mill (QM-3SP2, Nanjing University Instrument Factory, China), which had a 500 ml stainless steel vessel with 50 glass balls with diameters of 0.5–0.8 cm. Ball milling was conducted at room temperature with a frequency of 500 r/min for 3 h using a 3 min-milling and 1 min-idle mode to prevent overheating. After ball milling, the sample was collected for lignin extraction.

Lignin extraction was carried out based on a method described in the literature (Wen et al. 2013a). Briefly, 10 g of milled solid residue sample were extracted with 200 mL of 1,4-dioxane (96% by volume) at 100 ℃ for 1 h, followed by liquid/solid separation by vacuum filtration. The solid residues were further extracted with 200 mL 1,4-dioxane (96% by volume), followed by liquid/solid separation by filtration. The two extraction solutions were combined and concentrated to 60 mL using a rotary vacuum evaporator. The concentrated solution was slowly dropped into 180 mL of anhydrous ethanol to precipitate hemicellulose components. The precipitates were removed by centrifugation and the supernatant was further concentrated to 60 mL using a rotary evaporator. The concentrate was dropped into water with a pH of 2.0 (adjusted by hydrochloric acid) for lignin precipitation. The lignin precipitates were collected and freeze-dried. The dried lignin was further purified by dissolution into 90 wt% acetic acid solutions at a lignin/acetic acid mass (g)/volume (mL) ratio of 1:20. The lignin–acetic acid solution was dropped into water (hydrochloric acid, pH 2) for lignin precipitation. The lignin precipitates were centrifuged, collected and freeze-dried. The dried lignin samples were collected for further analysis.

### Lignin characterization

#### Gel permeation chromatography (GPC)

GPC was used to determine the contents of molecular weight of the lignin samples according to the method used in a previous publication (Wen et al. 2013b). Prior to GPC analysis, lignins were acetylated to improve their solubility in tetrahydrofuran (THF). Briefly, 125 mg lignin was dissolved in 7.5 mL of a solution of acetic anhydride: pyridine (1:1). After stirring for 24 h at room temperature in dark, the mixture was concentrated under reduced pressure by adding ethanol for several times. The mixture was dropped slowly into 200 mL of ice water (pH = 2.0) to induce precipitation, and the precipitate was washed with deionized water (3 × 50 mL). After centrifugation and freeze-drying, acetylated lignins were obtained. For GPC analysis, the acetylated lignins (4 mg) were dissolved in 2 mL of THF. The GPC analysis was conducted with an Agilent PL-GPC 220 (Agilent, USA) equipped with a refractive index detector (RID) on a PL-gel 10 μm Mixed-B 7.5 mm ID column. The analysis was carried out with THF as the mobile phase at a rate of 1.0 mL min^−1^ and injection volume of 10 μL. Calibration was performed using polystyrenes.

#### Two-dimensional nuclear magnetic resonance spectroscopy (2D ^1^H-.^13^C HSQC NMR)

Firstly, 80 mg of lignin was dissolved in 0.5 ml of DMSO-d_6_. The lignin solution was then transferred to a NMR tube with a diameter of 5 mm. The HSQC NMR spectra were acquired using a 400 MHz Bruker Avance NEO spectrometer at 25 °C using a standard Bruker pulse sequence ‘hsqcetgpsi2’. ^1^H spectra were acquired with a sweep width of 5000 Hz, 1024 sampling points, relaxation delay of 1.5 s. ^13^C spectra were acquired with a sweep width of 18,000 Hz, 256 sampling points and hydrocarbon coupling constant of 145 Hz. The Fourier transform process is performed prior to zero. The 2D HSQC spectra were processed using MestReNova software (14.0.0) and cross-peaks were assigned according to previous publications. NMR figures were prepared in Adobe Illustrator version 24.0.

#### Phosphorus-31 NMR (.^31^P-NMR)

31P-NMR was carried out to determine the contents of hydroxyl groups in lignin samples according to the method used in a previous publication (An et al. 2015). Briefly, 40 mg of lignin was dissolved in 500 μL of the mixture of anhydrous pyridine-d5 and CDCl_3_ (1.6:1, v/v) while being stirred. Then 100 μL of the cyclohexanol solution (10.85 mg mL^−1^ in the mixture of anhydrous pyridine-d5 and CDCl_3_ (1.6:1, v/v)) was added as the internal standard followed by the addition of 100 μL of a chromium (III) acetylacetonate solution (5 mg mL^−1^ in the mixture of anhydrous pyridine-d5 and CDCl_3_ (1.6:1, v/v)) as the relaxation reagent. Finally, the mixture was phosphitylated with 100 μL of TMDP for 10–15 min. Quantitative NMR spectra were acquired using an inverse-gated decoupling pulse sequence with a 30° pulse angle and a 4-s pulse delay, a 10-s relaxation time, and 256 scans. All NMR data were processed using MestReNova software (14.0.0).

### Biomass compositional analysis

Biomass composition (glucan, xylan, arabinan and lignin) of the untreated and pretreated corn stover samples were determined according to the standard procedure developed by the US National Renewable Energy Laboratory (NREL) (Sluiter et al. 2008).

### High performance liquid chromatography (HPLC)

HPLC was used to determine sugars and other biomass-derivatives generated in pretreatment and enzymatic digestion. For sugars analysis, the hydrolysates from pretreatment (obtained by filtration with filter paper from 20 g steam exploded wet corn stover mixed with 200 mL distilled water after being incubated at 50 °C and 180 r/min for 1 h) and enzymatic digestion were centrifuged at 12,000 rpm for 5 min and the supernatants were filtered through 0.22 μm filters before HPLC analysis. A Shimadzu HPLC system (LC-20AD) equipped with a refraction index detector (RID-10A, Shimadzu, Japan) and an Aminex HPX-87H column (Biorad, USA) were used to determine the sugars in the samples. The column temperature and detector temperature were controlled at 65 ℃ and 35 ℃, respectively. The mobile phase was H_2_SO_4_ (5 mM) with at a flow rate of 0.6 mL/min. In order to determine the total sugars in pretreatment hydrolysates, subsamples of pretreatment hydrolysates were hydrolyzed with 4% H_2_SO_4_ at 121 ℃ for 60 min. The hydrolyzed samples were processed using the same procedure as that for primary pretreatment hydrolysate prior to HPLC analysis (Gullon et al. 2010).

The non-sugars byproducts generated from pretreatment, such as furfural, 5-HMF and phenolic compounds (coumaric acid, 4-hydroxybenzadehyde, vanillin, syringaldehyde and ferulic acid) were analyzed using a HPLC system (LC-20AT) equipped with a UV/VIS detector (SPD-20A, Shimadzu, Japan) and an Agilent reversed phase TC-C18(2) column (250 mm × 4.6 mm) (Agilent, USA) was used. A wavelength of 280 nm was used for the detector with the column temperature controlled at 35 ℃. A gradient elution method was used with a mobile phase flow rate of 1.0 mL/min. The initial mobile phase was composed of 0.1% formic acid in water (Phase A) and 10% methanol in acetonitrile (Phase B). Phase B was increased from 0 to 10% from 1 to 15 min, and further increased to 35% from 15 to 20 min. Phase B was decreased from 35 to 10% from 20 to 30 min at which the cycle of the analysis was finished (Zhang et al. 2016a).

### Calculations

Glucan digestibility of and glucose yield from the pretreated corn stover samples were calculated based on the following equations:

1 $$Glucan \,recovery\, (\%) \,in\, solid\, residue\, after\, pretreatment\, =\, glucan \,content\, (\%) \,in \,solid\, residues \,\times \,biomass \,yield\, (\%) \,\times \,100$$

2 $$Glucose\, digestibility\, (\%) \,= \,total\, amount \,of \,glucose\, generated \,from\, enzymatic\, digestion\, \times\, 0.9/total \,amount\, of \,glucan\, in\, pretreated \,solid\, residue\, \times\, 100$$

3 $$Sugar\, yield\, (\%)\, = \,total\, amount\, of\, glucose\, or\, xylose\, in \,enzymatic\, or \,pretreatment \,hydrolysate/total\, amount \,of \,glucose\, or\, xylose\, equivalent\, in \,untreated\, corn \,stover\, \times\, 100.$$

#### Statistical analysis

All statistical analyses were performed using SPSS version 25 (SPSS, Inc., USA). Analyses of variance (ANOVA) followed by the least significant difference (LSD) test were performed to test the significance (*p* < 0.05) of the differences among the samples.

## Results and discussion

### Biomass composition and component recovery in solid residues after ICSE

In a previous study of the authors, a glycerol-assisted one-step ICSE was developed to pretreat corn stover at a corn stover/glycerol mass ratio range from 1:0.5 to 1:2.0 (Wang et al. 2020a). In that study, a stover/glycerol mass ratio of 1:0.5 led to the highest glucan digestibility though the highest glucose yield was achieved with the use of a stover/glycerol ratio of 1:1 (Wang et al. 2020a). Since glycerol is a low-cost carbon source and can be utilized by many microorganisms (Hassanpour et al. 2020b; Kao et al. 2013; Lubuta et al. 2019), further reducing glycerol loading would have the potential to develop an integrated enzymatic digestion and fermentation process without separation glycerol after ICSE pretreatment. In this study, the effect of glycerol loading on ICSE at a stover/liquid ratio of 1:0.5 was investigated. In addition, the effect of water extraction was also investigated since previous studies showed that extractives could affect pretreatment effectiveness (Li et al. 2016; Neves et al. 2016; Tajmirriahi et al. 2021).

Table 1 shows the biomass composition and component recovery in solid residues after ICSE at different mass ratios of glycerol/water with or without water extraction. Unextracted and untreated corn stover had relatively low contents of glucan, xylan and lignin as the contents of extractives and ash were relatively high (~ 30%). After water extraction, the contents of carbohydrates were improved though the glucan recovery was reduced due to the loss of soluble sugars in water extractives. Previously, it was reported that both water extractives and ethanol extractives could contribute to “lignin” content in biomass compositional analysis due to the condensation/precipitation of extractives (Thammasouk et al. 1997). As shown in Table 1, based on the standard NREL method, with which biomass composition was determined after both water and ethanol extraction, untreated corn stover had a lignin content of 14.9% while the use of water-extracted corn stover had a lignin content of 20.9%, corresponding to a lignin recovery of 114%. Since water extractives were removed, the increases in the recovery of “lignin” were likely from ethanol extractives during glucan and xylan determination at high temperature and acidic condition. It was previously reported that the ethanol extractives of corn stover were rich in phenolics (Li et al. 2016), though the contents and compositions of extractives varied among different biomasses (Li et al. 2016; Tajmirriahi et al. 2021; Thammasouk et al. 1997).

**Table 1:** Biomass composition and component recovery in solid residues after ICSE pretreatment

Water extraction prior to ICSE	G:W^2^	Biomass yield (%)	Content in solid residue (%)^3^			Recovery in solid residue (%)^4^		
			Glucan	Xylan	Lignin	Glucan	Xylan	Lignin
No	0:5	58.7	39.8 ± 1.5	12.9 ± 0.1	25.0 ± 1.0	75.1 ± 2.8	43.0 ± 0.3	98.5 ± 3.9
	2:3	62.8	39.9 ± 0.9	12.2 ± 0.2	26.4 ± 0.4	80.6 ± 1.8	43.5 ± 0.7	111.3 ± 1.7
	3:2	61.7	40.6 ± 0.4	12.1 ± 0.1	26.4 ± 0.8	80.5 ± 0.8	42.4 ± 0.4	109.3 ± 3.3
	4:1	61.1	39.9 ± 0.5	10.4 ± 0.2	28.6 ± 0.2	78.5 ± 1.0	36.1 ± 0.7	117.3 ± 0.8
	5:0	61.5	40.6 ± 0.4	12.3 ± 0.1	26.3 ± 1.1	80.3 ± 0.8	43.0 ± 0.3	108.6 ± 4.5
Yes^5^	0:5	54.6	43.4 ± 0.2	14.5 ± 0.1	23.5 ± 0.4	76.3 ± 0.4	45.0 ± 0.3	86.1 ± 1.5
	4:1	52.2	43.6 ± 0.4	12.6 ± 0.8	24.7 ± 0.2	73.2 ± 0.7	37.4 ± 2.4	86.5 ± 0.7
	Untreated	81.24	36.5 ± 0.1	20.6 ± 0.0	20.9 ± 0.6	95.3 ± 0.3	99.7 ± 0.0	114.0 ± 3.3
Corn stover determined by standard NREL procedure^5^		–	31.1 ± 0.3^7^	17.6 ± 0.2^8^	14.9 ± 0.9	–	–	–

1. All the trials were undertaken at solid/liquid mass ratio of 1:1 except for this one (no additional liquid); 2. G:W = glycerol:water (w/w); 3. the contents of arabinan (excluding water-soluble arabinan) were reduced to no more than 1.4% after pretreatment (data not shown); 4. component recovery in solid residue (%) = Component content (%) in solid residue × Biomass yield (%)/Component content (%) in untreated corn stover × 100; 5. hot water extraction at 90 °C for 2 h removed 18.8% water extractives (corresponding biomass yield of 81.2%); 6. untreated corn stover contained 18.4% water extractives, 5. 5% ethanol extractives and 6.4% ash (determined after both water and ethanol extraction based on the standard NREL procedure); 7. glucan content included 2.6% glucan equivalent (sucrose, glucose and fructose) from water extractives; 8. xylan content included 1.3% xylan equivalent

With the direct use of corn stover (no water extraction), ICSE pretreatment removed approximately 40% biomass components (corresponding biomass yields of ~ 60%) and led to slight increases in glucan content, substantial reductions in xylan content and significant increases in lignin content in the solid residues. The solid residues after pretreatment recovered 75–81% of total glucan and 36–44% of total xylan, respectively. ICSE at a mass ratio of glycerol to water of 4:1 led to the lowest content and recovery of xylan. These results indicated that at this condition, the extents of fiber softening and water penetration were balanced, leading to the maximal xylan removal. ICSE led to the recovery of more than 100% “lignin”, except for the pretreatment without glycerol (0:5). In the authors’ previous study in which corn stover/glycerol mass ratios of 1:0.5 and 1:2.0 were used, a high lignin recovery of 110% was also observed at corn stover/glycerol ratio of 1:0.5 (Wang et al. 2020a), which was similar with the lignin recovery in the present study. However, further increasing the mass ratio to 1:1 and 1:2 led to significant lignin recovery reduction to 99% and 81% (Wang et al. 2020a).

In contrast, the use of water-extracted corn stover for ICSE pretreatment led to reduced lignin recoveries in the solid residues compared to the direct use of the corn stover (no water extraction) (Table 1). These results indicated that the removal of water extractives did help to reduce the generation of new “lignin” or pseudo-lignin. The generated pseudo-lignin after ICSE of unextracted corn stover was likely due to the synergistic effects from water extractives, sugar degradation products and ethanol extractives. These pseudo-lignin products may have negative effects on sugar production by enzymatic digestion as demonstrated with different types of herbaceous biomass, such as rice straw, corn stover and sugarcane bagasse (Li et al. 2016; Neves et al. 2016; Tajmirriahi et al. 2021).

### Sugars, sugar derivatives and phenolics in pretreatment hydrolysates

Table 2 summarizes sugars and derivatives in pretreatment hydrolysates and estimates the mass balance. Overall, the levels of glucose equivalent in pretreatment hydrolysates derived from ICSE of unextracted corn stover were slightly higher than those of extracted corn stover, likely because the formers also contained C6 sugars from water extractives. There was no obvious trend on the levels of xylan equivalent in pretreatment hydrolysate as xylan and xylose were much less thermally stable than cellulose and glucose. The total glucan equivalents including those both in solid residues and pretreatment hydrolysates ranged from 82 to 89% with different pretreatment conditions while the total xylan equivalents ranged from 70 to 92%.

**Table 2:** Equivalents of glucan and xylan determined in pretreatment hydrolysates and total equivalents of glucan and xylan

Water extraction prior to ICSE	G:W^2^	Pretreatment hydrolysates							Total equivalent (%)^7^	
		Glucan equivalent (%)^3^			Xylan equivalent (%)^5^					
		Glucose	Gluco-oligoes^4^	Total	Xylose	Xylo-oligoes^6^	Furfural	Total	Glucan	Xylan
No	0:5	1.8 ± 0.0	5.4 ± 0.2	7.2 ± 0.2	4.4 ± 0.8	32.7 ± 1.2	0.6 ± 0.0	37.7 ± 2.0	82.3 ± 3.0	80.7 ± 2.3
	2:3	1.8 ± 0.2	6.6 ± 0.5	8.4 ± 0.7	6.4 ± 0.2	32.5 ± 0.3	1.1 ± 0.0	40.0 ± 0.5	89.0 ± 2.5	83.5 ± 1.2
	3:2	2.8 ± 0.1	5.1 ± 0.2	7.9 ± 0.3	6.8 ± 0.7	30.9 ± 0.4	1.1 ± 0.0	38.8 ± 1.1	88.4 ± 1.1	81.2 ± 1.5
	4:1	1.9 ± 0.2	5.5 ± 0.1	7.4 ± 0.3	6.7 ± 0.3	26.4 ± 0.3	1.1 ± 0.0	34.2 ± 0.6	85.9 ± 1.3	70.3 ± 1.3
	5:0	1.9 ± 0.2	5.7 ± 0.1	7.6 ± 0.3	6.0 ± 0.7	28.1 ± 0.6	0.6 ± 0.0	34.7 ± 1.3	87.9 ± 1.1	77.7 ± 1.6
Yes^1^	0:5	3.0 ± 0.0	2.5 ± 0.1	5.5 ± 0.1	3.7 ± 0.1	34.7 ± 0.4	0.5 ± 0.0	38.9 ± 0.5	90.1 ± 0.5^8^	91.9 ± 0.8^9^
	4:1	0.8 ± 0.1	4.6 ± 0.1	5.4 ± 0.2	2.1 ± 0.0	30.3 ± 0.2	0.9 ± 0.1	33.3 ± 0.3	87.0 ± 0.9^8^	78.7 ± 2.7^9^

1. For every 100 g corn stover, water extraction removed 2.6% glucan equivalent and 1.3% xylan equivalent; 2. G:W = glycerol:water (w/w); 3. glucan equivalent from HMF was negligible (≤ 0.03%); 4. gluco-oligoes included gluco-oligosaccharides and possible glyceryl glucosides; 5. xylan equivalent from furfural was not included (≤ 1.1%) as furfural could also generated from other C5 sugars; 6. xylo-oligoes included xylo-oligosaccharides and possible glyceryl xylosides; 7. total equivalent includes those both in solid residue (glucan or xylan recovery in Table 1) and pretreatment hydrolysate; 8. total glucan equivalent included those in solid residue, pretreatment hydrolysis and also glucan equivalent (sucrose, glucose and fructose) from water extractives; 9. total xylan equivalent also included those in solid residue, pretreatment hydrolysis and also xylan equivalent from water extractives

The phenolics in pretreatment hydrolysates after ICSE are summarized in Additional file 1: Table S1. Ferulic acid, coumaric acid, 4-hydroxy-benzaldehyde (HBA), syringaldehyde and vanillin were the major components detected in pretreatment hydrolysates by HPLC analysis. Coumaric acid and ferulic acid are hydroxycinnamic acids, which link lignin to polysaccharides through mainly γ-ester linkages with polysaccharides and *β*-O-4 ether linkages with lignin units (Lam et al. 2001). The total yield of ferulic acid and *p*-coumaric acid decreased with increasing the mass ratio of glycerol to water perhaps because of the *γ*-esterification reaction between hydroxycinnamic acids and glycerol and production of feruloyl glycerol and/or coumaroyl glycerol which prevents condensation of hydroxycinnamic acids on the surface of the fiber (Wang et al. 2020a). The yields of HBA, vanillin and syringaldehyde with the use of unextracted corn stover increased first with increasing glycerol loading then decreased, which was line with the observation in the authors’ previous publication (Wang et al. 2020a). HBA, vanillin and syringaldehyde can come from both ethanol extractives and lignin (Li et al. 2016). The yield variations of these phenolic compounds were the reflection of comprehensive reactions, including phenolics/lignin depolymerization and repolymerization as well as glycerol’s participation of in these reactions.

### Characteristics of lignin after ICSE

To understand the mechanism of the glycerol-assisted ICSE of unextracted and extracted corn stover, lignins extracted from enzymatic digestion residues were characterized.

#### Lignin molecular weight

Table 3 shows molecular weights of lignins extracted from enzymatic digestion residues. Glycerol-assisted ICSE reduced both weight-average molecular weight (M_W_) and number-average molecular weight (M_N_) (except for the M_N_ of lignin from ICSE of unextracted corn stover), indicating that glycerol addition reinforce the degradation of lignin and /or alleviated the condensation/repolymerization of lignin during ICSE. However, as shown in Table 1, this did not reduce the formation of pseudo-lignin in the ICSE of unextracted corn stover. Moreover, it was also observed that for the glycerol-assisted ICSE trials, the use of extracted corn stover led to the recovery of lignins with higher molecular weights (Table 3). It is generally believed that water extractives have buffering effects towards H^+^ ions from hot water, acetic acid from biomass and/or acid catalysts used in pretreatment (Li et al. 2016; Zhang et al. 2016b). The higher lignin molecular weights were likely related to lower pH and higher pretreatment severity in ICSE due to the removal of buffering water extractives, causing higher degrees of lignin condensation/repolymerization. These results also indicated the formation of pseudo-lignin due to the condensation/precipitation of extractives was not the (main) reason causing the increase of lignin molecular weight.

**Table 3:** Molecular weights of lignins extracted from enzymatic digestion residues

Water extraction prior to ICSE	G:W^1^	M_W_^2^ (g/mol)	M_N_^3^ (g/mol)
No	0:5	2977	937
	4:1	2838	951
Yes	0:5	3521	1040
	4:1	2851	955

1. G:W = glycerol:water (w/w); 2. M_W_, weight-average molecular weight; 3. M_N_, number-average molecular weight

#### ^1^H-^13^C HSQC 2D NMR spectra

Previously, it was reported that acid-catalyzed alcohol pretreatment led to the esterification at *α*-position and etherification at *γ*-position of aliphatic chains of lignin with alcohols (Dong et al. 2019; Hassanpour et al. 2020a). In order to investigate whether glycerol reacted with lignin in glycerol-assisted ICSE, ^1^H-^13^C HSQC 2D NMR analysis was undertaken before and after ICSE.

**Fig. 1.**
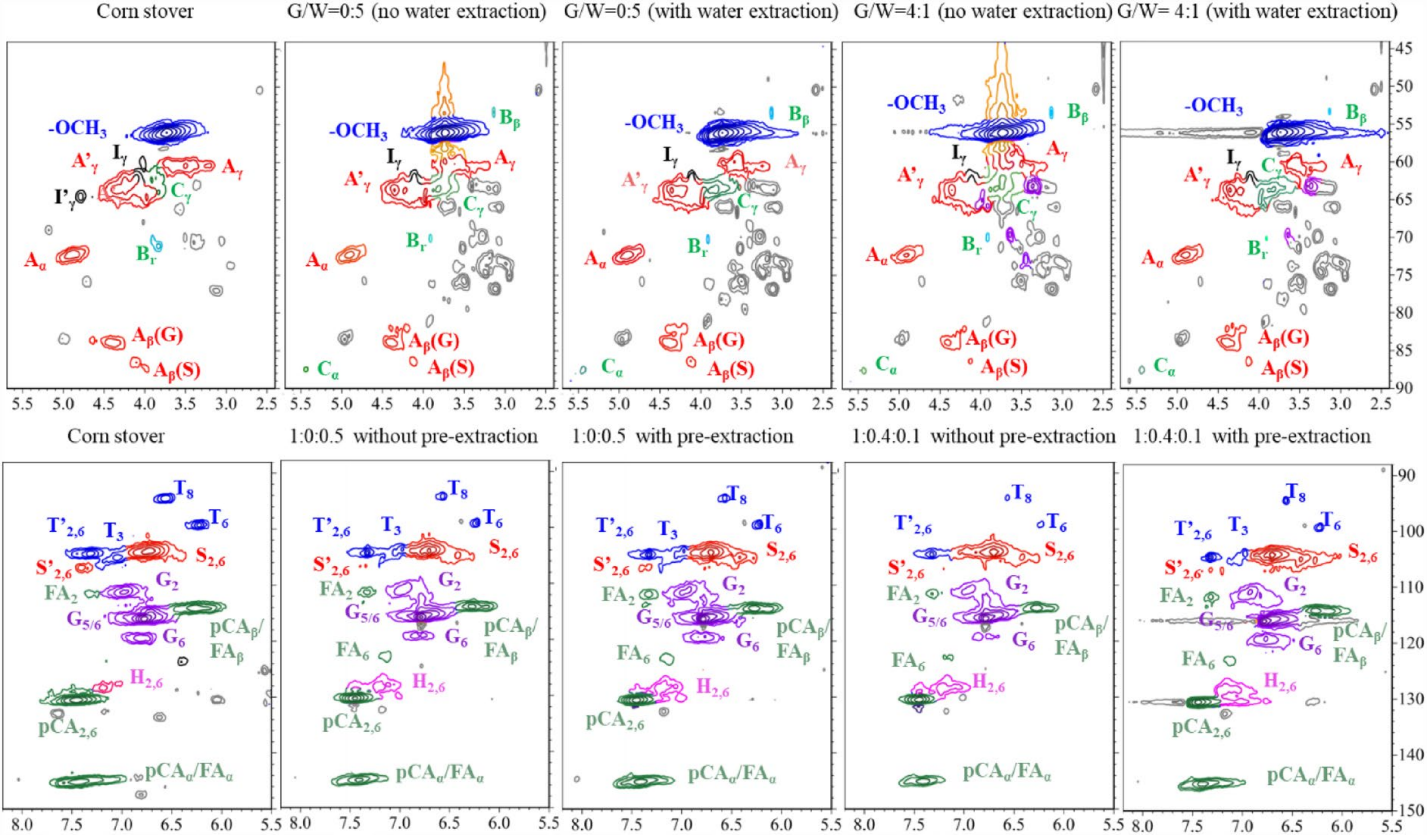
^1^H-^13^C 2D HSQC NMR spectra of lignin recovered from enzymatic digestion residues. G:W = glycerol:water (w/w)

Figure 1 shows the ^1^H-^13^C HSQC 2D NMR spectra of lignins. Signals at *δ*_C_/*δ*_H_ 53.4/3.74 and 57.87/3.74 (above and below the signal of -OCH_3_) were detected in the lignins derived from unwater-extracted and steam exploded corn stover, while they were absent in lignins derived from native corn stover, or water-extracted and steam exploded corn stover, which further indicated that water extraction before steam explosion could reduce the “pseudo-lignin” formation during steam explosion. Compared to glycerol-free ICSE, three additional signals at *δ*_C_/*δ*_H_ 62.6/3.4, 65.3/4.0 and 69.3/3.7 ppm were detected in the lignins derived from glycerol-assisted ICSE. These signals were related to the *γ*-esterification between hydroxycinnamic acids and glycerol (Hassanpour et al. 2020a). These signals also explained the low contents of ferulic acid detected in the pretreatment hydrolysates derived from the glycerol-assisted ICSE pretreatments compared to that from the glycerol-free ICSE (Additional file 1: Table S1) as ferulic acid was likely converted to feruloyl glycerol through esterification. The signal at *δ*_C_/*δ*_H_ 72.2/4.9 ppm related to the *α*-position of the *β*-O-4 linkages was present in all the lignin samples recovered from the enzymatic digestion residues. However, the signal related to the *β*-O-4 linkages etherified by glycerol, which was previously detected at *δ*_C_/*δ*_H_ 81.1/4.7 ppm in the acid-catalyzed glycerol pretreatment (Hassanpour et al. 2020a), was not detected (Fig. 1). These results indicated that etherification did not occur in glycerol-assisted ICSE, which was likely due to the relative high pH as external acid catalyst was not used in the glycerol-assisted ICSE.

#### Hydroxyl groups determined by ^31^P NMR

According to the ^31^P NMR spectra, the contents of hydroxyl groups of lignins were determined semi-quantitatively and shown in Table 4. Glycerol-assisted ICSE led to slight lower contents of aliphatic OH groups (excluding carboxylic OHs), further indicated that etherification at *α*-position of lignin aliphatic chain did not occur, which was different from the acid-catalyzed glycerol pretreament (Hassanpour et al. 2020a). Glycerol-assisted ICSE led to slightly higher contents of phenolic OH groups (from S, G & H units) and carboxylic OH groups in the lignins than glycerol-free ICSE, indicating intense degradation and /or lower level of condensation/repolymerization of lignin occurred in glycerol-assisted ICSE than in glycerol-free ICSE. Water extraction resulted higher amount of aliphatic OH and total phenolic OH, and lower amount of carboxylic OH in the lignins perhaps because of less pseudo-lignins formation, since pseudo-lignins had relatively lower amount of aliphatic hydroxyl groups and phenolic hydroxyl groups, and greater amount of carboxylic acid (He et al. 2018).

**Table 4:** Hydroxyl groups of lignins extracted from enzymatic digestion residues (mmol/g lignin)

Water extraction prior to ICSE	G:W	Aliphatic OH	S-OH	G-OH	H-OH	Total phenolic OH (S, G and H)	Carboxylic OH
No	0:5	2.06	0.34	0.41	0.67	1.42	0.83
	4:1	1.92	0.45	0.55	0.63	1.63	1.04
Yes	0:5	2.47	0.40	0.49	0.76	1.65	0.72
	4:1	2.24	0.47	0.53	0.77	1.77	0.79

G:W = glycerol:water (w/w); S-OH, syringyl OH; G-OH, guaiacyl OH; H-OH,* p*-hydroxylic phenyl OH

*β*-O-4 Linkages are the most abundant lignin internal linkages and account for up to 45–94% in lignins mildly isolated from plant materials (Munk et al. 2015). In biomass pretreatment, there are two possible mechanisms for the cleavage of *β*-O-4 linkages: heterolytic pathway and homolytic pathway (Obame et al. 2019). In the heterolytic pathway, a carbonium ion at the benzylic position can be formed in an acid-catalyzed pretreatment, which results in the cleavage of *β*-O-4 linkages and the formation of Hibbert ketones (Miles-Barrett et al. 2016; Obame et al. 2019). In the presence of alcohols, carbonium ions react with alcohols through etherification, which protects *β*-O-4 linkages from cleavage at relatively low pretreatment temperatures (Dong et al. 2019; Hassanpour et al. 2020a; Obame et al. 2019). However, in steam explosion pretreatments without additional acid catalysts, the heterolytic pathway may not be the dominant one because of relatively high pH and short incubation time (Obame et al. 2019).

In contrast, the homolytic pathway likely dominates in the cleavage of *β*-O-4 linkages in steam explosion, especially at high temperatures (e.g., 200 °C and above) (Heikkinen et al. 2014; Obame et al. 2019). Cleavage of *β*-O-4 linkages in the homolytic pathway leads to the generation of radicals and favors condensation/repolymerization through radial couplings (Heikkinen et al. 2014; Obame et al. 2019). In a recent publication, it was suggested that mannitol (a type of sugar alcohol) probably alleviated radical coupling (Chu et al. 2021a, 2021b). Like mannitol, glycerol might play a similar role in alleviating condensation/repolymerization though the mechanism is still unclear.

### Glucose production from ICSE-pretreated corn stover samples

#### Glucan digestibility

**Fig. 2:**
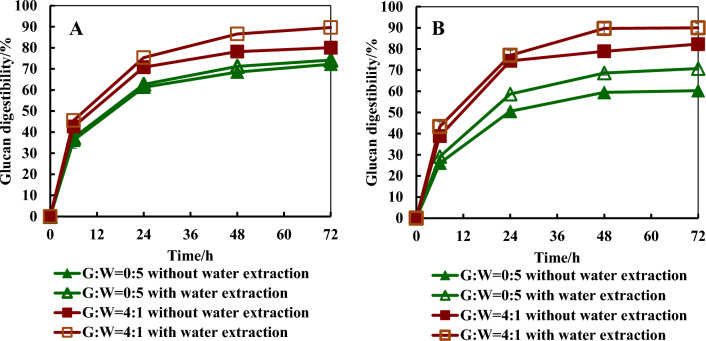
Glucan digestibility for representative enzymatic digestion of washed (**A**) and unwashed (**B**) pretreated corn stover. A, The pretreated corn stover was washed before enzymatic digestion; B, the pretreated corn stover was unwashed before enzymatic digestion; G:W = 0:5, corn stover was pretreated by ICSE with a solid:liquid ratio of 1: 0.5 and the liquid contained glycerol and water at a mass ratio of 0:5; G:W = 4:1, corn stover was pretreated by ICSE with a solid:liquid ratio of 1: 0.5 and the liquid contained glycerol and water at a mass ratio of 4:1; without water extraction, corn stover was unwater extracted before pretreated by ICSE; with water extraction, corn stover was water extracted before pretreated by ICSE

Table 5 summarizes glucan digestibility and sugar yield after 48 h enzymatic digestion as well as glucose yield increase compared to those from glycerol-free ICSE. Furthermore, Fig. 2 shows the digestion kinetics of the representative samples. With the use of unextracted corn stover, increasing the mass ratio of glycerol to water increased glucan digestibility till the ratio reached 4:1, at which the highest glucan digestibility of ~ 78% was achieved. After ICSE of unextracted corn stover, the use of unwashed solid residues led to similar glucan digestibility to those with the use of washed solid residues except for the samples achieved from the ICSE trials with glycerol/water mass ratios of 0:5 and 5:0. With glycerol-free ICSE, wash of pretreated stover improved glucan digestibility from 59.6% to 68.5%, 8.9% improvement. Regarding the digestibility of water-extracted and ICSE-pretreated corn stover samples, the use of washed corn stover from ICSE without glycerol addition led to a glucan digestibility of 71.2% and wash only slightly improved glucan digestibility. With the use of glycerol in ICSE at a glycerol/water mass ratio of 4:1, the glucan digestibility improved to 87–90% for extracted stover and wash did not increase glucan digestibility. The higher glucan digestibility of the solid residue derived from glycerol-assisted ICSE of water-extracted corn stover than that of the solid residue from unextracted corn stover was likely attributed to less pseudo-lignin formation due to the removal of water extractives.

**Table 5:** Glucan digestibility and glucose yield from enzymatic hydrolysate per 100 g corn stover with different conditions

Water extraction prior to ICSE	G:W^1^	Digestibility, %		Glucose yield, g/100 g corn stover		Relative glucose yield increase (%)^2^	
		Washed	Unwashed	Washed	Unwashed	Washed	Unwashed
No	0:5	68.5 ± 2.6	59.6 ± 3.0	17.8 ± 1.2^3^	18.0 ± 0.2	0.0	1.1 ± 1.1
	2:3	72.0 ± 6.4	69.5 ± 4.0	20.0 ± 1.6	22.2 ± 0.9	12.4 ± 9.0	24.7 ± 5.1
	3:2	73.1 ± 3.0	72.4 ± 0.6	20.3 ± 1.0	22.9 ± 0.4	14.0 ± 5.6	28.7 ± 2.2
	4:1	78.2 ± 2.0	78.1 ± 1.4	21.2 ± 0.6	23.7 ± 0.3	19.1 ± 3.4	33.1 ± 1.7
	5:0	76.3 ± 1.6	72.5 ± 0.8	21.1 ± 0.6	22.7 ± 0.3	18.5 ± 3.4	27.5 ± 1.7
Yes	0:5	71.2 ± 1.1	68.6 ± 1.0	20.3 ± 0.2	20.7 ± 0.3	14.0 ± 0.6	16.3 ± 1.7
	4:1	86.6 ± 1.0	89.7 ± 2.5	23.6 ± 0.5	25.5 ± 0.6	32.6 ± 2.8	43.3 ± 3.4

1. G:W = glycerol:water (w/w); 2. relative glucose yield increase (%) = (glucose yield at each condition—glucose yield at reference condition) × 100/glucose yield at reference condition; 3. reference condition

The above results indicated that water extraction reduced the negative effect of pretreatment hydrolysates on glucan digestion of corn stover pretreated by ICSE without glycerol addition. Li et al. (2016) and Tajmirriahi et al. (2021) reported that water extractives itself had less effect on cellulose digestibility and glucose yield (Li et al. 2016; Tajmirriahi et al. 2021). Thus, we speculate that the inhibitory effect of pretreatment hydrolysates on enzymatic digestibility was likely related to the compounds generated from ICSE of unextracted corn stover. The presence of water extractives promoted the formation of these inhibitory compounds though water extractives were not the only precursors. Furthermore, the presence of glycerol likely softened the fibers, making the fiber deconstruction easier. In the authors’ previous publication, it was reported that glycerol-assisted ICSE produced corn stover with higher roughness (Wang et al. 2020a), increasing the cellulose accessibility and glucan digestibility. Moreover, pseudo-lignin derived from water extractives during glycerol-assisted ICSE and deposited on the surface of the pretreated residues had adverse impact on glucan enzymatic digestibility. Therefore, removal of water extractives led to increased glucan digestibility of the solid residue from glycerol-assisted ICSE.

Previous studies found that sugars, aldehydes and phenols in pretreatment hydrolysates could inhibit enzymatic digestion (Jonsson and Martin 2016; Liu et al. 2016b; Mhlongo et al. 2015; Qin et al. 2016; Zhai et al. 2018; Zhu et al. 2016). Moreover, for solvent-based pretreatments, residual solvents, such as ionic liquids could also have negative effects on enzymatic digestion (Liu et al. 2016a). Removal of inhibitors by wash is an effective approach to enhance glucan digestibility but can consume and generate large volume quantities of organic-rich water streams. Treatment of organic-rich water streams and recovery of solvents from the dilute solution can be a significant cost component of the whole process.

**Fig. 3:**
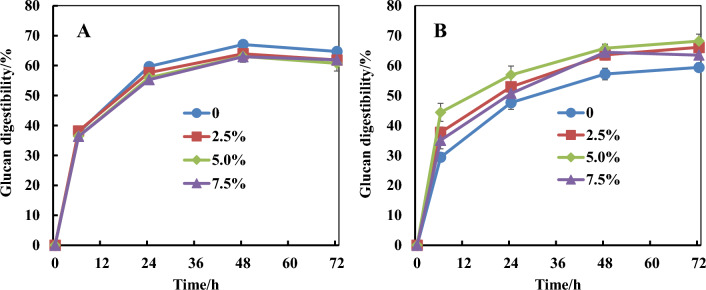
Effect of glycerol addition on glucan digestibility of un-water-extracted corn stover pretreated by glycerol-free ICSE (stover/glycerol/water ratio of 1:0:0.5). **A** Corn stover was washed before enzymatic digestion; **B** corn stover was unwashed before enzymatic digestion

For the solid residues generated from glycerol-assisted ICSE, wash did not cause significant change to glucan digestibility except for the one with the highest glycerol/water mass ratio of 5:0. In order to investigate the role of glycerol, samples derived from glycerol-free ICSE of unextracted corn stover were hydrolyzed with or without glycerol addition. As shown in Fig. 3, glucan digestibility was slightly inhibited with the use of washed biomass in the presence of 2.5% to 7.5% glycerol. This observation was in line with a previous study (Zhang et al. 2015), in which addition of glycerol inhibited enzymatic digestion of washed sugarcane bagasse following acid-catalyzed glycerol pretreatment. Interestingly, glucan digestibility was improved with the use of unwashed biomass in the presence of 2.5 to 7.5% glycerol, indicating glycerol might alleviate the inhibitory effect of pretreatment hydrolysates on cellulase. Glycerol, like other polyols (xylitol, sorbitol, trehalose and sucrose) may protect protein structure from the first hydration sphere by preferential exclusion, thereby increasing the energy required for unfolding and improving protein stability (Liu et al. 2010). In addition, as reported previously, glycerol may react with glycose or xylose through glycosylation to form glyceryl glycosides during ICSE which can act as enzyme stabilizer for glucan enzymatic digestion (Sawangwan et al. 2010; Zhang et al. 2013).

The glucan digestibility of washed solid residue after glycerol-assisted ICSE of unextracted corn stover at a stover/glycerol/water ratio of 1:5:0 was 76% in the present, 23% lower than that achieved at the same pretreatment condition in the authors’ previous study (Wang et al. 2020a). The higher glucan digestibility achieved in the previous study was likely due to the use of different batch of corn stover. In addition, a high cellulase loading of 20 FPU/g dry substrate (~ 50 FPU/g glucan) was used in the previous study compared to the use of 20 FPU/g glucan in the present study.

Since cellulase Ctec3 is robust in hemicellulose hydrolysis, xylan digestibility and xylose yield were also calculated after 48 h enzymatic digestion for each treatment. Water extraction before steam explosion with or without glycerol also could increase xylan digestibility and xylose yield of unwashed pretreated corn stover, although less effectiveness was observed for that of the washed pretreated ones. However, unlike cellulose enzymatic hydrolysis, glycerol inhibited xylan enzymatic hydrolysis by cellulase Ctec3 because xylan digestibility and xylose yield decreased with the increase of glycerol concentration (Additional file 1: Table S2). The mechanism needs to be further illustrated.

#### Glucose yield

Table 5 also summarizes the total sugar yields at different conditions. For glycerol-free ICSE, although wash improved glucan digestibility, it did not improve total sugar yields since pretreatment hydrolysates contained both monosugars and sugar oligomers. For glycerol-assisted ICSE of unextracted corn stover, the use of unwashed corn stover led to the higher yields of sugars. Increasing glycerol/water ratio from 2:3 to 4:1 significantly increased glucan digestibility and glucose yield of unwashed solid residues (*p* < 0.05). Compared to the glucose yield of the washed solid residue derived from glycerol-free ICSE of unextracted corn stover, glycerol-assisted ICSE improved glucose yield by 12%-19% with washed solid residues and by 25%-33% with unwashed solid residues. For glycerol-assisted ICSE of water-extracted corn stover, glucose yield of unwashed solid residue was slightly higher than that of washed solid residue. Water extraction further enhanced glucose yield by 32.6% and 43.3% with washed and unwashed residues (G:W = 4:1), respectively.

As shown in Additional file 1: Table S3, at a glycerol/water mass ratio of 4:1, ICSE of unextracted corn stover, followed by enzymatic digestion of unwashed solid residue at 3% glucan loading generated a medium containing 36.2 g/L glycerol, 29.0 g/L glucose and 5.1 g/L xylose. In contrast, ICSE of water-extracted corn stover, followed by enzymatic digestion produced a medium containing 27.9 g/L glycerol, 32.3 g/L glucose and 7.2 g/L xylose. It should be noted that the variation in glycerol was due to less glycerol was used for the water-extracted materials since glycerol loading was based on the weight of solid for ICSE. Such media may be directly used for fermentation by many microorganisms, such as *Rhodosporidium toruloides*, *Yarrowia lipolytica*, *Clostridium pasteurianum*, to produce bioproducts, such as microbial oils, mannitol, citric acid and n-butanol (Hassanpour et al. 2020b; Kao et al. 2013; Lubuta et al. 2019).

## Conclusions

The present study investigated the effects of water extraction, glycerol loading and wash on the effectiveness of ICSE and glucan digestion of corn stover for fermentable sugar production. The results showed that ICSE of unextracted corn stover led to substantial formation of pseudo-lignin compared to ICSE of water-extracted corn stover. In the glycerol-assisted ICSE, lignin modification only occurred at *γ*-position of aliphatic chains through esterification. Glycerol removal of corn stover derived from glycerol-assisted ICSE pretreatment was not necessary and ICSE pretreatment of raw corn stover at glycerol/water mass ratio of 4:1 likely was the optimal condition because of the highest glucan digestibility and relatively high glucose yield. Glycerol-assisted ICSE of water-extracted corn stover produced more glucose compared to the use of unextracted raw corn stover. Enzymatic digestion of corn stover from glycerol-assisted ICSE pretreatment without wash led to the production of hydrolysates rich in glucose and glycerol, which may be directly used to produce biological products by many microorganisms.

### Supplementary Information

**Additional file 1**: **Table S1.** Phenolics produced from corn stover after being pretreated with glycerol reinforced steam explosion (mg/100 g corn stover). **Table S2.** Xylan digestibility and xylose yield from enzymatic hydrolysate per 100 g corn stover with different conditions. **Table S3.** Glycerol and sugars in enzymatic hydrolysate at 3% glucan loading (g/L).

## Data Availability

The datasets used and/or analyzed during the current study are available from the corresponding author on reasonable request.
