# Supplementary material for: Enhanced enzymatic sugar production from corn stover by combination of water extraction and glycerol-assisted instant catapult steam explosion
Source: Bioresour Bioprocess. 2024 Mar 14;11(1):31. doi: 10.1186/s40643-024-00739-7 (PMC10992945; doi:10.1186/s40643-024-00739-7)
Supplement: Supplementary file 1 — Additional file 1: Table S1. Phenolics produced from corn stover after being pretreated with glycerol reinforced steam explosion (mg/100 g corn stover). Table S2. Xylan digestibility and xylose yield from enzymatic hydrolysate per 100 g corn stover with different conditions. Table S3. Glycerol and sugars in enzymatic hydrolysate at 3% glucan loading (g/L). [file 40643_2024_739_MOESM1_ESM.docx]

**Additional Materials**

**Insights into water pre-extraction and glycerol-assisted** **instant catapult steam explosion of corn stover for efficient enzymatic digestibility without glycerol removal**

Fengqin Wang^a^, Hongli Dong^a^, Weiwei Yu^a^, Yinling Gao^a^, Guotao Mao^a^, Yanxia An^d^, Hui Xie^a,*^, Andong Song^a,^*, Zhanying Zhang^b,c^

^a^ Key Laboratory of Enzyme Engineering of Agricultural Microbiology, Ministry of Agriculture, College of Life Science, Henan Agricultural University, Zhengzhou 450002, PR China

^b^ Centre for Agriculture and the Bioeconomy, Queensland University of Technology, 2 George St, Brisbane, QLD 4000, Australia

^c^ School of Mechanical, Medical and Process Engineering, Faculty of Engineering, Queensland University of Technology, 2 George St, Brisbane, QLD 4000, Australia

^d^ College of Food Science and Technology, Henan Agricultural University, Zhengzhou 450002, PR China

*Corresponding author: Hui Xie

Phone: +86-371-68555260

E-mail: xiehui@henau.edu.cn

*Corresponding author: Andong Song

Phone: +86-371-56552976

E-mail: [songandong@henau.edu.cn](mailto:songandong@henau.edu.cn)

Table S1 Phenolics produced from corn stover after being pretreated with glycerol reinforced steam explosion (mg/100g corn stover).

| Water extraction prior to ICSE | G:W | Ferulic acid | *p*-Coumaric acid | HBA^1^ | Vanillin | Syringaldehyde |
| --- | --- | --- | --- | --- | --- | --- |
| No | 0:5 | 13.3±0.6 | 18.5±0.8 | 27.8±2.0 | 22.6±1.2 | 58.7±15.3 |
|  | 2:3 | 11.5±0.2 | 17.0±1.3 | 35.3±0.6 | 25.3±0.4 | 50.4±6.6 |
|  | 3:2 | 10.1±1.1 | 16.7±1.6 | 37.2±4.1 | 36.6±1.3 | 74.5±7.2 |
|  | 4:1 | 7.5±0.3 | 16.6±0.8 | 31.0±1.3 | 23.4±2.4 | 75.7±0.5 |
|  | 5:0 | 5.5±0.5 | 15.6±3.4 | 23.1±1.0 | 18.7±2.6 | 50.7±3.6 |
| Yes | 0:5 | 17.5±1.1 | 9.2±0.0 | 30.5±5.6 | 21.6±0.9 | 87.3±3.7 |
|  | 4:1 | 15.9±0.8 | ND^2^ | 27.0±1.4 | 23.2±1.2 | 75.9±1.8 |

1. HBA: 4-Hydroxybenzaldehyde; 2. ND, not detected; 3. G:W=Glycerol : Water (w/w).

Table S2. Xylan digestibility and xylose yield from enzymatic hydrolysate per 100 g corn stover with different conditions

| Water extraction prior to ICSE | G:W^1^ | xylan digestibility, % | |  | xylose yield, g/100 g corn stover | |
| --- | --- | --- | --- | --- | --- | --- |
|  |  |  |  |  |  |  |
|  |  | Washed | Unwashed^2^ |  | Washed | Unwashed |
| No | 0:5 | 40.8±2.3 | 40.3±0.9 |  | 3.5±0.2 | 7.0±0.1 |
|  | 2:3 | 41.2+0.7 | 29.1±0.8 |  | 3.6±0.1 | 5.7±0.1 |
|  | 3:2 | 39.8±0.5 | 25.7±0.8 |  | 3.4±0.1 | 5.1±0.0 |
|  | 4:1 | 41.1±1.2 | 22.7±0.8 |  | 3.0±0.0 | 4.2±0.1 |
|  | 5:0 | 43.3±0.6 | 21.5±1.5 |  | 3.7±0.1 | 4.3±0.1 |
| Yes | 0:5 | 38.9±0.7 | 53.4±1.1 |  | 4.1±0.1 | 7.3±0.2 |
|  | 4:1 | 38.9±1.2 | 39.2±1.0 |  | 3.4±0.1 | 4.8±0.1 |

1. G:W=Glycerol : Water (w/w); 2. Xylan digestibility = (xylose yield from enzymatic hydrolysate - xylose yield from pretreated hydrolysate)*0.88/(xylan content in solid residues + xylan content in pretreated hydrolysis) * 100.

Table S3 Glycerol and sugars in enzymatic hydrolysate at 3% glucan loading (g/L)

| Water extraction prior to ICSE | G:W | Glycerol | Glucose | Xylose |
| --- | --- | --- | --- | --- |
| No | 0:5 | No glycerol | 23.1±0.3 | 9.0±0.1 |
|  | 4:1 | 36.2±0.5 | 29.0±0.4 | 5.1±0.1 |
| Yes | 0:5 | No glycerol | 25.2±0.3 | 11.1±0.2 |
|  | 4:1 | 27.9±0.1 | 32.3±0.8 | 7.2±0.1 |

Note: G:W=Glycerol : Water (w/w).
